# Educating the nurses of tomorrow: exploring first-year nursing students’ reflections on a one-week senior peer-mentor supervised inspiration practice in nursing homes

**DOI:** 10.1186/s12912-024-01768-5

**Published:** 2024-02-20

**Authors:** Daniela Lillekroken, Heidi M. Kvalvaag, Katrin Lindeflaten, Tone Nygaard Flølo, Kristine Krogstad, Elisabeth Hessevaagbakke

**Affiliations:** Department of Nursing and Health Promotion, Oslo Metropolitan University, PB 4 St. Olavs plass N, 0130 Oslo, Norway

**Keywords:** Clinical practice, Content analysis, Focus groups, Learning transfer, Nursing education, Nursing homes, Nursing students, Peer mentoring

## Abstract

**Background:**

Worldwide, the healthcare system stresses a severe deficit of nurses because of elevated levels of work-induced stress, burnout and turnover rates, as well as the ageing of the nursing workforce. The diminishing number of nursing students opting for a career in nursing older people has exacerbated this shortage. A determining factor in the choice of a career within the field of residential care for nursing students is educational institutions offering students learning opportunities with positive learning experiences. Therefore, educational institutions must develop programmes that employ student active learning methods during clinical periods. Although much focus has been given to the development of new educational programs, insufficient consideration has been given to the value of peer mentoring and students’ interactions during the clinical placement at nursing homes. The aim of the present study is to explore first-year nursing students’ perceptions and experiences with peer mentoring as an educational model during their inspiration practice week at nursing home.

**Methods:**

The study employed a qualitative exploratory and descriptive research design. Data collection took place in October 2022 using focus group interviews. A total of 53 students in their first year of the bachelor’s programme at the Oslo Metropolitan University participated in eight focus group interviews. The data were analysed following the principles of inductive content analysis.

**Results:**

The analysis resulted in one main category, ‘Being inspired—keep learning and moving forward’, representing first-year nursing students’ common perceptions of being mentored by third-year students. The main category is supported by two categories: ‘Closeness to the mentor’ and ‘Confidence in mentors’ professional knowledge and teaching and supervision methods’, which are interpreted as the drivers that enabled first-year students to learn more about nurses’ roles and responsibilities in the nursing home.

**Conclusion:**

Mentorship enhances the learning transfer from third-year nursing students over to first-year nursing students by providing them with real-world exposure and guidance from their more experienced peers. This hands-on approach allows them to bridge the gap between theory and practice more effectively, boosting first-year nursing students’ confidence and competence in nursing and caring for older people living in nursing homes.

## Background

Nursing is one of the main professions that provides care to older people [1]. To meet society’s challenges of providing quality healthcare to older people, knowledgeable and skilled future generations of nurses are needed [2]. International research reveals that one of the key challenges for nursing in residential care is recruiting and retaining knowledgeable and skilled nurses [3]. Although nursing students have positive [4], or moderately positive attitudes towards nursing older people [5], they generally do not see caring for older people as an interesting area of their future careers [6]. Students may lack the motivation to study and work in this field; therefore, it is necessary to increase the attractiveness of working within the gerontological nursing field [7].

Generation Z nurses, born 1995 or later (aged ≤ 24 years of age), have introduced new expectations and ideals of life and work into the nursing profession [8]. People belonging to generation Z exhibit traits such as tolerance, respect, social-change oriented, collaboration and confidence but with caution while embracing diversity and growing up with friends from various ethnic backgrounds [9, 10]. To meet their expectations and retain them into the nursing profession, it is vital to design educational programmes and work conditions accordingly. Moreover, to ensure that graduating nurses possess the necessary levels of gerontological nursing competence, nursing education programmes must prepare future nurses accordingly. This implies that faculties must emphasise the importance of having gerontological nursing knowledge and competences among nursing students right from the early years of training [11]. This may contribute to providing comprehensive education to nursing students and instil a positive attitude towards nursing older adult patients [7].

Nursing education in Norway, as well as in other European countries, complies with the European Union’s (EU) directives [12, 13], and is completed in accordance with the Bologna Process [14], requiring bachelor’s and master’s degrees as the norm. This means that it takes 180 ECTS (European Credit Transfer System) to obtain a bachelor’s degree and a further 120 ECTS to complete a master’s degree. In Norway, nursing education consists of at least 4,600 h, including theoretical knowledge and clinical practice, in which clinical practice represents half of the education period; therefore, clinical practice must cover a minimum of 2,300 h [12]. As required by the EU [12, 13], theoretical and clinical studies alternate during these three years, and students intertwine theoretical and clinical knowledge during lectures, seminars, workshops and clinical periods conducted in different clinical contexts. After attending a three-year nursing education programme, the student achieves a bachelor’s degree in nursing as a registered nurse (RN) with competence at a general level. For students to obtain a nursing degree, they must demonstrate the knowledge and ability required in the national goals to become RNs at the end of their education, consisting of three main goals: knowledge, skills and general competence [15].

Since 2020, Oslo Metropolitan University [OsloMet], as well as other Norwegian universities, has implemented a new bachelor’s programme in nursing. The programme aims to qualify candidates for practicing professional nursing based on up-to-date evidence-based knowledge, professional suitability and respect for human autonomy and participation [16].

To educate knowledgeable and skilled nurses to meet Norwegian society’s healthcare challenges, knowledge and skills of how to provide better and safer fundamental care are part of the curriculum of the first year during the bachelor’s programme in nursing [15], and clinical placements in nursing homes where students learn to plan and provide fundamental care to older people are mandatory courses [12, 17]. During the course ‘Theoretical Foundations of Nursing’ (SYK1000) that is taken in the students’ first term, the first-year students have a one-week clinical period (inspiration practice) in nursing homes. This one-week inspiration practice period is in addition to their six-week clinical placement during the second term. The focus of the inspiration practice is to observe and gain knowledge about the nurse’s role and responsibilities in nursing homes, including planning and participating in providing fundamental care to nursing home residents. During this period, the third-year nursing students attend the clinical period ‘Nursing Patients with Complex Health Challenges’ (SYKPRA60) in nursing homes. One of the learning outcomes of this course is related to students developing skills and knowledge about learning, mastering and changing processes, as well as supervising and teaching patients, next-of-kin, students and healthcare personnel. To pass the clinical period, as a mandatory learning activity, the third-year students will supervise, plan and carry out supervision for one or a group of two to three first-year students in cooperation with the nurse preceptor and nurse educator from the university [16], hence employing peer mentoring as a learning and teaching method during the clinical period at nursing home for both student groups.

Mentoring is an encouraging and supportive one-to-one relationship with a more experienced worker or peer student and is characterised by positive role modelling, promoting aspirations, positive reinforcement, open-ended counselling and joint problem-solving [18]. Peer mentoring is a relational process where a more experienced individual (mentor) contributes to the professional and personal development of a less experienced individual (mentee) [19]. This approach aligns with the educational philosophy of peer-assisted learning, which engages students in the teaching process [20]. However, it is worth noting that the term ‘peer mentoring’ lacks a consistent definition [21]; therefore, various interchangeable terms, such as ‘peer learning’, ‘peer coaching’ and ‘near-peer teaching’, are utilised in the literature [22]. In the present study, ‘peer mentors’ or ‘mentors’ refers to senior nursing students possessing more extensive experience than their junior counterparts, the ‘mentees’, and ‘peer mentoring’ refers to the process of learning transfer from mentors to their mentees.

The inspiration practice period has been implemented to provide first-year students with insights into the nurse’s role and responsibilities in nursing homes, hence, to prepare them for their first clinical placement period at nursing home and all subsequent clinical periods throughout their education. This preparation aims to prevent the occurrence of what is termed ‘reality shock’ [23], a phenomenon that may lead to negative consequences for their continuing nursing education and influence their choice of whether to pursue a career in nursing [24].

Despite the growing number of studies revealing the importance of the professional development of nursing students in clinical studies, little is known about the peer mentoring process used by students in learning from each other in higher education [25]. Results from previous studies reveal that peer mentoring increases mentees’ integration, academic success, class retention, self-esteem, psychosocial wellness, reduces anxiety in clinical setting, increases self-worth for both the mentee and the mentor [26–30]. Furthermore, positive outcomes for mentors have been observed, ranging from enhanced problem-solving abilities to heightened coping skills [31, 32]. Recently, results from a longitudinal study indicate that a one-on-one mentorship program is beneficial for the retention of new graduate nurses, particularly during the first year [33, 34].

Learning environment quality in clinical placement is vital for how nursing students achieve competence through reflection on their experiences [35]. Similarly, positive learning experiences in residential care are vital for their future choices regarding where to work and therefore crucial for employers striving to recruit newly qualified nurses. Facilitating optimal clinical mentoring is therefore of high priority in nursing education [36].

As shown above, although peer mentoring has been reviewed in many studies, several gaps on the effects the mentor program has in the context of nursing home as teaching and learning context remain. Specifically, no programs focus mentoring on a targeted discipline or degree of interest to cultivate specific gerontological professional development. Because of this, there is a lack of literature focusing on the first-year experience of a nursing student. Likewise, there is limited available research exploring the benefits of mentoring specifically for first-year nursing students during the clinical placement at nursing homes as a learning context. Therefore, the aim of the present study is to explore first-year nursing students’ perceptions and experiences with peer mentoring as an educational model during their inspiration practice week at nursing home.

## Theoretical framework

To the best of the researchers’ knowledge, the application of peer mentoring as a learning and teaching strategy for first-year students within the context of nursing home learning is a novel approach. Therefore, the application of innovative and active learning strategies in clinical settings necessitates educational research. For the present study, the theory of learning transfer described by Wahlgren and Aarkrog [37] was chosen as the theoretical framework. The theory of transfer of learning is defined as the application or adaptation of previously learned knowledge, skills or understanding to new situations or contexts. Moreover, it involves the ability to make connections and use what a student has learned in one context to solve problems or understand concepts in different contexts. However, little is known about the processes used by students to transfer learning from each other and to apply or adapt knowledge to practice.

The theory of transfer of learning is influenced by three factors that may be seen as facilitators or barriers that promote or hinder students’ learning in clinical settings: (i) person-related transfer factors, which include motivation, the ability to set goals, having confidence and knowing how to apply the new knowledge and reflecting on how to apply the new knowledge [38]; (ii) teaching-related transfer factors, which refer to how the ‘teacher’ organises the learning situation, by, for example, giving theoretical and examples and demonstrating how to apply theoretical knowledge into real-life situations [38]; and (iii) factors related to the situation where the knowledge is applied [37], such as the context of where the knowledge is applied, that is, willingness to include the workers’ new knowledge and skills in the workplace, leadership characterised by openness to positive changes and willingness of using the necessary resources. During the analysis, the content of the processes described by students when learning from each other revealed similarities with the theory of transfer of learning [37]; therefore, the researchers decided to choose this theory as a framework for discussing the study’s findings.

## Methods

### Aim of the study

This study aims to explore first-year nursing students’ perceptions and experiences with peer mentoring as an educational model during their inspiration practice week at nursing homes.

### Study design

The present study has a qualitative exploratory descriptive design [39]. The design was appropriate because it allowed the researchers to contextualise how the first-year students perceived peer mentoring and nursing home as learning environment and their role as mentees within the context of nursing home, thus providing a picture of what naturally occurred between the mentors and mentees.

### Study setting

The study was conducted at Oslo Metropolitan University during the one-week inspiration practice at nursing homes for first-year nursing students.

### Study population and sampling

All the students enrolled in the first year of the bachelor’s programme in nursing at the Department of Nursing and Health Promotion in the academic year 2022–2023 were informed about the study and invited to participate. All six researchers were engaged in providing information about the study and in the process of recruiting potential participants.

The students were provided with verbal and written information about the study during a face-to-face first meeting before and after inspiration practice week. For inclusion, the students should: (i) be enrolled in the academic year 2022–2023, (ii) voluntary to attend the study, (iii) agreed to be recorded during the interviews. If the students were interested and expressed their wish to participate, they were asked to contact the researchers by email and agree upon the date for the interview. When distributing the participants in focus groups, to make the participants feel confident and comfortable during the interviews, the researchers considered the students’ class affiliation and formed groups with students belonging to the same class, thus fostering a sense of familiarity and ease among the participants.

Of a total of 488 students enrolled in the academic year 2022–2023, only 53 expressed their interest and agreed to participate. The ages of the participants ranged between 19 and 54 years. Although most had no work experience in the field of healthcare/nursing, some had up to 13 years of clinical experience working in nursing homes or home care. The researchers strived to provide a gender balance among the participants; therefore, an equal proportion of female and male participants was encouraged to participate. Even so, only seven participants were males. As the research literature has demonstrated, nursing is a female-dominated profession with individuals still choosing gender role stereotypes for their careers [40, 41] This may explain the large number of females among the participants.

### Data collection

Data were collected during the fall semester of 2022, one week after the students conducted their inspiration practice week. Eight focus group interviews were conducted to collect data during October– November 2022. Focus groups involve people with similar characteristics coming together in a relaxed and permissive environment to share their thoughts, experiences and insights [42]. The choice of using focus group interviews as data collection methods was because allows participants share their own views and experiences, but also listen to and reflect on the experiences of other group members [42]. This synergistic process of group members interacting with each other promotes and refines participants’ viewpoints to a deeper and more considered level and produces data and insights that would not be accessible without the interaction found in a group [42, 43]. Prior to conducting the interviews, a semistructured interview guide inspired by peer mentoring in nursing literature was developed and used to guide the interviews. The interview guide used in the present study was developed based on recommendations from previous studies for further research to achieve a comprehensive understanding of how peer mentoring can be effectively employed in the context of nursing home [22, 23, 26]. The themes and questions that were posed during the interviews are presented in Table 1.

**Table 1:** Interview guide

Themes	Questions
**Opening questions**	1. Do you have any prior experience from working in nursing homes? 2. How many days did you spend in the nursing home as part of the Inspiration practice course? 3. How did you experience the Inspiration practice course in the nursing home?
**Information and preparation period**	1. How would you describe the preparation period and information you received from your supervisor at OsloMet? 2. Do you have any suggestions for improvements?
**Reception at the nursing home**	1.How would you describe the first day on placement in the nursing home and the welcome you received from the third-year students?
**Quality of supervision**	1. How would you describe the peer mentoring teaching and learning method you received from the third-year students? Can you provide some examples? 2. How would you describe your expectations of the supervision from the third-year students, and to what extent were these expectations met? Can you provide some examples? 3. Do you have any suggestions for improvements?
**Peer mentoring as a teaching and learning method in clinical setting**	1. What was it like to be supervised by third-year students? 2. In your opinion, what are the advantages/challenges of being supervised by third-year students? 3. Can you provide some suggestions for potential improvement for the third-year students’ peer mentoring as a teaching and learning method?
**Learning environment**	1. How did you perceive nursing home as a context to learn about the provision of nursing care? 2. Can you describe what contributed to your learning and, if applicable, any challenges you encountered that hindered learning? 3. Can you please describe experiences that contributed to your perception of the Inspiration practice course as useful for learning the role and responsibilities of a nurse? 4. Do you have any suggestions for improvements?
**Knowledge of the nurse’s role and responsibilities in nursing homes**	1. Can you please describe the values that characterize the nursing profession in a nursing home context? 2. Can you please provide examples of where you see the importance of applying theoretical knowledge in the practice of nursing in a nursing home? 3. In your opinion, what can influence the development of good observation skills, the so called “clinical gaze”? 4. How would you describe the nurse’s role and responsibilities in a nursing home? 5. The main goal of the Inspiration practice course was to gain insight into the nurse’s role and responsibilities in a nursing home. What impression do you have of what a nurse in a nursing home does? 6. How would you describe your observations of the interaction between nurse and patient during the Inspiration practice week? Can you provide some examples? 7. What tasks does the nurse perform and which responsibilities does the nurse have for patients, relatives, and staff? 8. In which way has the Inspiration practice week prepared you for the next clinical placement in a nursing home?
**Learning outcomes**	1. In your opinion, was the Inspiration practice course useful? 2. Can you please describe something specific that you have learned during this week? 3. What would you describe as important for you to get the most out of this course?
**Closing questions**	1. Is there anything you would like to add, emphasize, or comment on before we finish? 2. Any final remarks? Thank you for sharing your experiences!

The number of participants in each focus group ranged between 3 and 12. Depending on the number of participants in each focus group and on their verbal dynamism during the interviews, each focus group interview lasted between 30 and 55 min. The focus group interviews were held in a quiet classroom after a seminar class. As recommended by Krueger and Casey [42], the researchers planned to conduct each focus group interview in pairs. However, because of the busy work schedules among researchers, only two focus group interviews were conducted by two researchers, one acting as a moderator and the other as a ‘secretary’. While the moderator’s role was to pose questions and follow up the answers, the secretary’s role was to take notes, observe the group dynamic and use the recording device. During the interviews, the participants were encouraged to talk openly, share their thoughts and experiences with one week of inspiration practice in a nursing home and offer suggestions for improvement for the course. Hence, the participants offered deep and rich answers that contributed to the detailed expression of opinions.

### Data analysis

All eight focus group interviews were digitally recorded and transcribed verbatim by the researchers immediately after completion. Except for one researcher (KK) who transcribed four focus group interviews, all authors transcribed each one to two focus group interviews. However, depending on the length of the interviews and the richness of the dialogs, the transcription process lasted between 6 and 8 weeks. The data generated from eight focus group interviews consisted of 106 A4 pages taped with 1.5 line spacing and Times New Roman font size. The analysis process has additionally taken eight weeks.

When conducting a focus group interview, it is the group rather than the individual that is the focus of analysis because data generated from focus groups represents situated accounts that can provide in-depth insights into contextualised social interactions [43]. The transcripts from the interviews were analysed following the three steps of inductive content analysis outlined by Kyngäs [44]: preparation, organising and reporting the findings.

As part of the first step, data analysis began during data collection through careful group moderation. By following transcription, reflexive engagement with the data enabled researchers’ familiarity with it as a whole before the coding process. The empirical data generated from eight focus groups were analysed independently by two researchers (DL & HK) to identify the key categories coded onto transcripts. At this step, the coding process helped reduce the amount of data. These codes were subsequently subjected to a more detailed subcoding of meaningful content, such as one word or a shorter sentence. At this step, no theoretical understanding influenced the selection of the units of analysis. Unit selection was based on the themes from the interview guide and derived from the data. Both authors then met and discussed the similarities and differences between the coded data from each interview, sharing their overall understanding of the data. If discrepancies occurred, they were solved by discussing before making a final decision.

In the second step, the researchers discussed, analysed and decided which codes should be grouped together into subcategories and determining the hallmarks of the categories. Following a discussion about the open coding process, a coding tree was developed to facilitate comparisons within and between groups. To validate and maximise the trustworthiness of the initial findings, a descriptive overview of the final analysis was presented to the other researchers, that is, the coauthors of the present paper, to confirm that it was a realistic interpretation of their views. For example, the code ‘following the mentors everywhere’ has gradually been incorporated into the subcategory ‘Spending time with mentors.’ In this step, influenced by the learning transfer theory [37] this subcategory was further placed under a category labeled ‘Closeness to the Mentor.’ It was interpreted as a person-related factor that facilitates learning transfer, thereby inspiring first-year students to continue learning and moving forward.

The third step was to present the findings by describing the content of the subcategories and categories as supported by participant quotes. An example of the coding tree is shown in Table 2.

**Table 2:** Example of coding tree

Codes	Sub-categories	Categories	Main category
‘…following the mentors everywhere…’ ‘I observed how my mentor changed a stoma bag…’ …’They were very open and receptive…’ ‘They explain in an easier way…’	Spending time with mentors Perceiving the mentors as role models Feelings of insecurity Mutual learning– learning from each other.	Closeness to the mentor	Being inspired– keep learning and moving forward
‘She had so much knowledge…’ ‘They provide us with answers’ ‘…they communicate with us by using professional terms…’ ‘very good to communicate with the residents’ ‘They also encouraged us to ask questions…’ ‘they [mentors] asked us if we would do anything different’ ….’they had a good plan for us…’	Mentors’ theoretical and practical knowledge and skills Mentors’ ability to apply diversity in didactical and pedagogical methods	Confidence in mentors’ professional knowledge and teaching and supervision methods	

### Rigour of the study

Rigour was ensured by employing several strategies. First, to ensure trustworthiness and rigour, the criteria described by Lincoln and Guba [45], known as credibility, dependability, confirmability and transferability, were employed.

To ensure transferability and dependability, the researchers clearly described the study’s theoretical framework, the recruitment and the characteristics of the participants, the research context, data collection and analysis processes so that readers could assess whether findings were applicable to their specific contexts and, if desired, repeating the study.

The data analysis was iterative and continued until all members of the research team agreed on a relevant and trustworthy formulation of the categories. To enhance trustworthiness, the consistency and dependability of data analysis was optimised by researcher triangulation. Two members of the research team (DL & HK), who independently coded interview transcripts and managed the coding and developed categories and subcategories that were assessed, verified and amended by all the members of the research team. Discrepancies in the coding were resolved through discussions until a consensus for each interview transcript was reached.

Confirmability is ensured by researchers presenting quotes from the participants that support the findings. The researchers strived to accurately represent the information provided by the participants, hence indicating that the interpretations of the data were not invented or based on preconceived notions.

In qualitative research, reflexivity should be oriented towards personal, interpersonal, methodological and contextual issues in the research [46]. Personal reflexivity refers to researchers reflecting on and clarifying their expectations, assumptions, and conscious and unconscious reactions to contexts, participants, and data [46]. The research team was composed of six women, all of whom had teaching experience with and knowledge of the first-year curriculum. Five of the research team members had experience with designing and conducting qualitative studies and collecting and analysing qualitative data. Although the analysis was performed by two researchers, all the researchers brought important contextual knowledge and insights to the analysis discussion, thus strengthening the study’s dependability. However, the researchers’ professional backgrounds as nurse educators who had knowledge of the curriculum and the course’s expected learning outcomes could address certain topics or follow-up questions during the focus group interviews, thus influencing the answers. Therefore, to minimize bias, the researchers discussed their prior experiences with interviewing, reflected on how questions were asked, and simultaneously managed their assumptions around how participants thought about and experienced being in the one-week inspiration practice.

Interpersonal reflexivity refers to the existing relationships and power dynamics between researcher and participants [46]. The participants in this study were first-year students, and some of the researchers who conducted the interviews were their teachers. Consequently, during the interviews, the power balance between researchers and participants could result in participants feeling that they were being evaluated, potentially leading to a focus on more positive experiences. To avoid this, researchers reinforced to participants that their participation is voluntary and that their answers will not influence their study progression. Moreover, during the interviews, researchers encouraged quieter participants to answer and allowed for differences of opinion.

Methodological reflexivity refers to researchers critically consider the nuances and impacts of their methodological decisions [46]. To strengthen methodological reflexivity, researchers discussed whether the study’s aim aligns with the chosen design and whether the data collection method and interview guide will generate data to answer questions posed during the focus group interviews. Another method to enhance methodological reflexivity was discussing the theoretical framework’s relevance to the study. After considerable discussions, the researchers decided to choose the theory of learning transfer [37] as it was considered the best theory to inform the data.

Contextual reflexivity entails researchers understanding the unique setting of the study [46]. To strengthen the study’s contextual reflexivity, researchers discussed which aspects of the context could influence the research and people involved, as well as how the research impacts the context. The study was conducted at a Norwegian university, and participants were enrolled in the first year of the nursing bachelor’s program. Although the interview guide was inspired by previous literature on peer-mentoring, the questions posed were developed to gain knowledge about students’ experiences with a one-week inspiration practice at a nursing home. This means that the research was influenced by the curriculum and mandatory courses conducted at this university. During discussions, some researchers mentioned that most focus group participants reflected on their clinical development and were looking forward to their turn being a mentor for first-year students. It was evident that this study also had a positive impact on participants.

### Ethical approval

The present study was granted approval to be conducted from the researchers’ institution, Department of Nursing and Health Promotion at Oslo Metropolitan University and from the Norwegian Agency for Shared Services in Education and Research (Sikt/Ref. number 334855). The study was conducted in accordance with the Helsinki Declaration [47]. Informed consent, consequences and confidentiality were all obtained and maintained. All participants received verbal and written information about the study and written informed consent was obtained from all the participants prior to data collection. The participants were also informed that they would not receive any financial or other benefits for participating in the study. All participants were assured that, should they choose to withdraw from the study at any time and for any reason, there would be no negative consequences for their education at the university. Nevertheless, the researchers were mindful of the students’ potential vulnerability due to their role as students, which might discourage them from withdrawing. However, despite no reported discomfort during interviews, the potential for discomfort or reluctance to express negative experiences exists. Therefore, before each focus group interview, the students were reminded of their option to withdraw from the interview, providing them with additional opportunities to assent to or withdraw from the study. None of the students who agreed to be interviewed reported any discomfort during the interviews, and none chose to withdraw.

### Findings

Following data analysis, one main category was generated, ‘Being inspired—keep learning and moving forward’, which was interpreted as the first-year nursing students’ common perception of being supervised by third-year students for one week of inspiration practice at nursing homes. During the interviews, the first-year students mentioned several times that they perceived third-year students as their mentors. To differentiate between first-year students and those in their third year, the third-year students will be referred to as ‘mentors’ throughout the manuscript.

Two categories—(i) ‘Closeness to the mentor’ and (ii) ‘Confidence in mentors’ professional knowledge and teaching and supervision methods’—were interpreted as the drivers enabling first-year students to learn more about nurses’ roles and responsibilities in nursing homes. Each category is supported by several subcategories.

In the following section, the findings are presented with excerpts from the participants’ statements. The statements end with a number representing the code each participant (i.e., P1) and focus group (i.e., FG2) were given before conducting the focus group interviews, meaning participant 1 in focus group 2.

### Closeness to the mentor

This category was supported by four subcategories: spending time with mentors, perceiving mentors as role models, feelings of insecurity and mutual learning– learning from each other.

### Spending time with mentors

The first subcategory was related to the time first-year students spent with their mentors. Because the mentors could allocate more time to spending with the first-year students, this time allowed mentors to share formal and informal knowledge and create learning opportunities for first-year students. Being close to the mentor and spending time together was decisive for several first-year students to experience a positive relationship with their mentor. This positive mentor-first-year student relationship was highlighted as one of the participants’ positive experiences in the inspiration practice. They experienced that their mentors were aware of their own roles and responsibilities and encouraged first-year students to follow them everywhere to gain insights into how it is to be a nurse employed at a nursing home. One of the participants said the following:

We were following the mentors everywhere… They explained us everything… However, we were only six students at that nursing home, so we get one mentor each… and I followed my mentor all the time, and she explained me a lot about how to help the resident with personal hygiene or how to use a Hoyer lift to help the resident to move from bed to wheelchair. I feel that I learned a lot.… (P4, FG1).

Other first-year students were grateful that, by being with mentors, they had the opportunity to be introduced to more complicated procedures, such as changing a stoma bag or measurements of vital signs or even weighing the residents. One participant shared her experience:

Yes, we have experienced a lot! We contributed to making breakfast and served it, we helped residents with personal hygiene… we weighed the residents and documented in their journal, and we learned how to document everything we did to or with a resident, in generally… However, I learned a new word: stoma and… [stoma bag]. I observed how my mentor changed the stoma bag to a resident. You know, I get the opportunity to meet the residents face-to-face and the life at that ward. (P1, FG3)

The first-year students stated that, with this type of supervision, they would be much more likely to reach their learning outcomes for the inspiration practice. One of the participants stated the following:

I feel that, for me, everything was good. They [mentors] showed us that they have knowledge… they were very open and receptive if we had some questions: ‘Just ask me!’ and they were honest if they could not provide the answer. It wasn’t like at school: ‘Use the contact form’ [laughter]… we got the answer at once, so this was OK. They were also very creative. They made cases about things we already had knowledge about, and I learned to use several measurement instruments, such as QSOFA [Quick Sepsis Related Organ Failure Assessment] and this kind of thing.… (P1, FG8).

### Perceiving mentors as role models

The second subcategory was related to first-year students perceiving the mentors as role models. Being close to the mentor, the first-year students could engage in informal discussions, hence finding that mentors were people who had been in their shoes, who had journeyed close to where they wanted to be and who had made their own mistakes in their learning but also gained practical knowledge. They perceived mentors as someone who was close enough to them, willing to share their wisdom and experience, and could help them avoid certain pitfalls. These perceptions contributed to developing a positive relationship with the mentors, which positively influenced their learning. One of the participants said the following:

I am happy that my first encounter with practice was through third-year students. It is not a long time since they were in our situation, so they know how it feels. They explain in an easier way… and you get a kind of insider information… yes, they provide us with information that nurses don’t say because they believe that we already know things… I think that because they were in this situation, they explain or teach us things in the same way they wish they have been told… They have established good routines for learning to achieve learning outcomes.… (P3, FG5).

### Feelings of insecurity

The third subcategory was related to feelings of insecurity among first-year students. Several first-year students asserted that they were not confident when they had to help the residents with their fundamental needs, such as toileting, changing diapers, personal hygiene or eating and drinking. One of the participants shared her experience:

I have never assisted someone with personal hygiene before… It was quite an experience…I felt hesitant, but I had to manage somehow… (P4, FG2).

Being close to the mentor offered opportunities to seek support. They appreciated that mentors accepted their insecurity, lack of experience and theoretical knowledge limitations. One of the participants said the following:

Going together with my mentor, I felt safe to fail… [laughter]. I am happy that I gained the opportunity to try and experience the challenges that came with… They asked questions and they sensed that we were not sure about the answer, but we gradually became confident when they ‘pushed’ us to try it on our own.… (P3, FG6).

### Mutual learning– learning from each other

The last subcategory was related to the learning process as a mutual process. Some of the first-year students had clinical experience in healthcare services as healthcare assistants. This placed expectations on the inspiration practice period, and although these students knew the field very well, they were impressed by the amount of practical knowledge they gained during this week. However, being close to the mentor offered opportunities to learn from each other. When the mentors could not answer their questions, they experienced that they searched for knowledge and together agreed about the correct answer for the given situation. The participants experienced that learning was a mutual process, and it did not happen only from mentors to them but also vice versa, as one of the participants said:

Yes, we had a positive dialogue about knowledge… sometimes it was funny to see… I think that it was a positive experience for both of us [to share knowledge], that when we asked questions, they had to search for the answer… and figure it out together… This would not happen with a nurse that has 20 years’ experience that knows the answer: ‘that is it!’… (P1, FG4).

### Confidence in mentors’ professional knowledge and teaching and supervision methods

This category was supported by two subcategories: mentors’ theoretical and practical knowledge and skills, and mentors’ ability to apply diversity in didactical and pedagogical methods.

### Mentors’ theoretical and practical knowledge

The first subcategory relates to the first-year students’ perceptions of mentors’ professional competence, which can be defined in theoretical knowledge, skills and general competence. The first-year students were positively surprised about their mentors’ amount of theoretical and practical knowledge. This contributed to motivating first-year students to be curious and wanting to learn more. Several first-year students asserted that their expectations for the inspiration practice week were fulfilled because of the supervision they gained from mentors, hence assessing mentors as ‘competent’, meaning ‘knowledgeable and skilled’. One of the participants said the following:

I was quite content with my mentor… She [the mentor] had so much knowledge… it seemed that she worked there [at nursing home] for 10 years… I was motivated by that because I noticed how much they [mentors] have learned during these three years.… (P3, FG6).

Other first-year students reported that they got answers no matter what they asked. They were surprised by the mentors’ theoretical knowledge and how they could provide them with examples of the application of theory in real patient situations. This contributed to an increase in first-year students’ self-confidence. One of the participants described his experience as follows:

Our mentors were very knowledgeable and skilled… They provide us with answers… I was surprised how much knowledge a third-year student could gain through education… As third-year students, they were so well prepared to work and to meet patients in the clinical field.… (P10, FG5).

Other participants were impressed by mentors using professional language during formal and informal conversations and by the clinical gaze they developed. One participant stated the following:

… and they communicate with us by using professional terms… such as… I don’t remember all of them now, but they [mentors] mentioned frontal lobe, and other [laughter]… and yes, ‘she’s got Alzheimer’s [referring to a nursing home resident]… it’s only a name for me… but, you know, Alzheimer’s means that the woman has dementia… (P5, FG7).

The mentors’ practical skills were also praiseworthy among first-year students. They observed and learned from mentors how to use different medical instruments and measure vital signs/National Early Warning Score (NEWS) or the level of haemoglobin or insulin on real patients and then documenting the results. One participant said the following:

I could see that they [the mentors] were knowledgeable and skilled… when they presented and demonstrated for us, they knew what they were doing and talking about… They taught us and demonstrated different measures, and when we asked them, they answered us… yes, they were professional.… (P2, FG7).

A skill that first-year students could easily perceive as a challenge was communication with residents who had a cognitive impairment. However, several first-year students were impressed by the mentors’ communication skills. Many were surprised by the ethical challenges imposed by communication with people with dementia. Others noticed how respectful mentors were when asking the residents for permission to bring into the resident’s room another person who would assist the resident with personal hygiene or toileting. One of the participants expressed this as follows:

He [the mentor] I had was very good at communicating with the residents… he always asked them if we could enter the room to observe or help with the provision of personal hygiene.… (P2, FG8).

### Mentors’ ability to apply diversity in didactical and pedagogical methods

The second subcategory was related to first-year students’ perceptions of the mentors’ ability to teach and supervise them and the diversity in didactical and pedagogical methods employed. The participants were content with the mentors’ explanations and demonstrations of all the work tasks a nurse has during a working day at a nursing home. Because the first-year students were not aware of what they should ask about, they particularly liked when their mentors provided them with knowledge without being asked for it or just demonstrated how the medical instruments or personal lift-assist device functioned. For most of them, this was perceived as the most appreciated first-hand knowledge, which mentors ‘just shared’ with them. They were also encouraged to ask questions and eventually provided additional answers if they could. One of the participants explained this as follows:

When we asked the mentors ‘Why are doing in this way and not in another…’, they always had good answers grounded in theory or in their prior clinical experiences… They acted very confident, so we also felt confident in what we were doing.… (P5, FG1).

Most of the participants were content with mentors’ methods of teaching or supervising them and giving feedback. They appreciated when mentors supported and encouraged them to learn things and become independent, but also to try new things and teach them how to do it. They appreciated being told what and how to help the resident prior to entering the resident’s room, not just being told what they had to do while the resident observed and listened, thus making them uncomfortable (i.e., during the provision of personal hygiene for a resident). One participant shared his less positive experience with providing personal hygiene to a female resident:

I had to ask my mentor how I should wash her body, and when I came to her breasts, I became very uncomfortable, but the mentor said to me, ‘Just lift her breasts and wash under and dry gently… it is OK’, and then I did it, but it was a strange experience.… (P3, FG7).

Another participant gladly shared her positive experience of being taught different procedures and routines regarding hygiene routines:

We had an interesting overview of hygiene routines at the ward, and then, we went through infection control equipment, and we had to take on and off, to learn these routines… We also learned how many times, how and when we had to use disinfecting alcohol on our hands and the order of taking on and off all that infection control equipment… a kind of ‘learning by doing’… (P1, FG2).

Another learning method that was much appreciated by first-year students was mentors asking questions during a procedure that engaged first-year students to reflect on knowledge before answering. One participant said the following:

When we got out of the resident’s room, they [mentors] asked us if we would do anything different.… (P3, FG7).

Because of the limited number of nursing homes that could have both first- and third-year students at the same time in the clinical field, a few of the first-year students had to complete their inspiration practice week by being two or three days at school or/and the department’s simulation learning environment and only one or two days in the nursing home. Although these students expressed that they learned a lot from their mentors, their expectations for inspiration practice week were not as positive as they expected to be. Some asserted that they got limited or almost no insights into the nurse’s role and responsibilities in the nursing home. One of the participants revealed her experiences in the department’s simulation learning environment:

Together with a few other students from my group, we were at the school’s simulation environment… They [mentors] had a good plan for us. The first day began with measuring vital signs on each other. and we could do it many times. They created several patient cases where we could measure and document NEWS for each case… Then, we learned to change the sheets on the bed while a ‘patient’ was lying there… I felt that I learned a lot, and I am content with how mentors taught us different procedures; however, I wish I could have been at a nursing home because, personally, I have no clinical experience; it would have been useful to get insights into the nurse’s role and responsibilities at nursing home before we start the clinical period at nursing home.… (P3, FG6).

During the focus group interviews, those first-year students who completed the inspiration practice week at the school’s simulation learning environment revealed some learning and teaching methods employed by their mentors, asserted as being very creative. The mentors could not offer learning activities regarding some procedures that could be done in real life (i.e., changing wound dressing on a resident’s leg ulcer); therefore, they had to think outside the box and create situations that could contribute to learning. One of the participants explained this as follows:

They [mentors] drew a ‘wound’ on their own leg and, by following the procedure, they changed the wound dressing on each other to demonstrate us how to change a leg ulcer dressing. I have to say that I learned a lot, although the wound was ‘fake’… [laughter]. (P2, FG7)

## Discussion

The aim of the present study was to explore first-year nursing students’ perceptions and experiences with peer mentoring as an educational model during their inspiration practice week at nursing homes. The analysis of the empirical data revealed that first-year students were inspired by their mentors, an inspiration that contributed to their learning progression.

As the findings have revealed, as a learning process, peer mentoring facilitates the transfer of learning by mentors designing instructional activities, thus encouraging first-year students to make connections between the theoretical knowledge they gained at school and the simulation learning environment and practical knowledge within new and real patient situations.

The findings from the current study have revealed first-year students’ descriptions of how mentors provided them with explicit instructions on how to apply knowledge or skills, thus engaging them in problem-solving activities that required learning transfer. Through these instructions, the mentors transferred learning over to first-year students, hence enabling their reflective thinking within the context of a nursing home. Moreover, acting as role models, being available and allocating time to be together with first-year students, the mentors were perceived as knowledgeable and skilled, features that contributed to enhancing first-year students’ motivation to search for new and more knowledge and, thus, to achieve learning outcomes. These features can be understood as person-related factors, which Wahlgren and Aarkrog [37] described as one of the factors facilitating learning transfer. Moreover, a person-related transfer factor was positively related to those participants who had previous clinical experience. As the findings have revealed, if the mentors could not answer the questions, the experienced participants, based on their previous clinical experience, suggested solutions; thus, learning was transferred the other way around, from the first-year students to mentors, with learning perceived as a mutual process [48].

In the present study, the first-year students showed receptiveness to acquiring knowledge and were concerned with making the most of the inspiration practice week. Their interest in learning was strengthened by mentors’ knowledge and abilities in providing instructions. This finding is similar to and supports the findings from previous studies demonstrating that peer mentoring contributes to students’ engagement and increases their cognitive skills, self-confidence, autonomy, clinical skills and reasoning [22, 49, 50].

The mentors’ specific knowledge about nurses’ roles and responsibilities in nursing homes, different procedures and communication challenges with people with cognitive impairment enhanced trust and the credibility of mentors’ preparedness for inspiration practice week. This led to first-year students’ trust in mentors’ ability to transfer learning. The participants’ curiosity and desire to gain insights into real-life patient situations have enabled their willingness to engage in learning activities. In the current study, the mentors adopted an active role when teaching and supervising first-year students. As the participants described, the mentors gladly shared their knowledge, demonstrated how to perform procedures and had informal and formal discussions about how first-year students could implement theory into practice. Similar to previous studies, which have demonstrated that learning with an equal peer facilitates making friends and developing relationships [25], hence reducing nursing student anxiety in the clinical setting [29] and promoting learning, the findings from the current study have revealed that the participants leaned on their mentors and felt safe and could trust their mentors. Although a few felt uncomfortable being exposed to new challenges (i.e., providing personal hygiene or helping residents with toileting), most of the participants stated that the mentors’ feedback given both during and postprocedure performance contributed to increasing their self-confidence when performing measures of vital signs or other procedures. These features resonate with Wahlgren and Aarkrogs’ [37] teacher-related transfer factor which emphasises the mentor’s ability to organise learning situations by including demonstrations, providing examples from theory and practice and reflecting on possible applications in real-life patient situations.

As suggested above, although person- and teacher-related transfer factors facilitated transfer learning, the situation-related factor raised some challenges. Despite the results from one study [51] demonstrating that nursing homes as a clinical placement will not add something new to students’ skills and competencies required for their future practice, other studies [35, 52] have demonstrated that, in general, learning in a clinical context can affect nursing students’ learning outcomes and satisfaction, as well as influence their choice of future career. Although simulation may prepare students for clinical learning environments, there is no comparison to the learning that comes from nursing patients in a real clinical context and from a simulation learning environment at school [53].

The findings from the current study revealed that not all the students were content with the learning context during their inspiration practice week. Some first-year students, together with their mentors, used the department’s simulation learning environment and even classrooms as a learning context for two or three days or even for the entire week. In this situation, it is reasonable to think that situation-related transfer factors [37] posed some challenges, and they were not related only to mentors’ pedagogical methods, but also to the programme’s readiness to inspiration practice week and the leadership of the related factors of the nursing home (i.e., not being able to provide enough placements). If the first-year students and their mentors had the necessary theoretical knowledge but could not apply it in a real-life patient situation, the person-related transfer factors could also be challenged. Although none of the participants expressed that using the department’s simulation learning environment as a learning environment was worthless, some hinted at their disappointment. The lack of situational transfer factors seemed to negatively affect the participants’ motivation to gain knowledge. However, as the participants asserted, their mentors’ creativity contributed to creating potential patient situations similar to those in real life. They also encouraged first-year students to simulate different patient conditions and perform different procedures, thus creating opportunities for first-year students to apply theoretical knowledge and improve their skills. This supports the idea that, despite a lack of situational transfer factors, the transfer of learning was supported by mentors’ teacher-related transfer factors rather than situational transfer factors.

Finally, being a first-year student supervised by knowledgeable and skilled third-year students can contribute to first-year students mirroring themselves and their knowledge with their peers. Thus, first-year students can become more aware of themselves as professionals and develop an understanding of the nurse’s role and responsibilities in the nursing home. Consistent with results from previous studies, the results of the present study suggest that peer mentoring facilitates the development of self-understanding in students [25, 26, 32, 36], which is essential for first-year students to gain a positive attitude towards nursing older people. The findings from the present study have suggested the use of peer mentoring in nursing education with structured training and supervision. Moreover, as the findings have indicated, peer mentoring facilitates learning transfer from mentors to mentees and provides valuable leadership experience for third-year students as mentors. In addition, mentoring may enhance a first-year student’s opportunity to be mentored and provide mentoring in the future.

### Implications for nursing education and clinical practice

Peer mentoring, as a teaching and learning method, can be applied to enhance nursing curricula and clinical practice in several ways. Firstly, incorporating successful peer mentoring strategies into the curriculum can foster a collaborative and supportive learning environment among nursing students. The perceived closeness between mentors and first-year students suggests that fostering strong mentor– first year student relationships can serve as a driver for effective learning in the context of nursing homes. This closeness may create an environment that facilitates open communication, trust, and a sense of support, which are essential elements in the field of nursing. Additionally, the confidence instilled in first year students regarding their mentors’ professional knowledge and teaching and supervision methods can directly impact the students’ understanding of nurses’ roles and responsibilities in nursing homes. In clinical practice, the findings from the study can be used to promote mentorship programs that facilitate knowledge transfer and skill development among nurses and among senior and novice students during their clinical periods. Lastly, the study highlights first-year students’ overall positive experiences with peer mentoring program. This positive experience can help change students’ attitudes towards nursing older people, making it an interesting aspect of their future careers.

### Strengths and limitations

The present study has several limitations that must be considered when interpreting the findings. First, although many students were invited to participate, the study was limited by a relatively small sample size restricted to students from Oslo Metropolitan University, hence limiting the findings’ national and international transferability. However, one strength may be that the findings and issues raised are relevant for both national and international nursing education programmes that apply the peer mentoring teaching and learning model in clinical placements. Another limitation may be the sample size and data saturation. As a concept, data saturation in qualitative research has been subject to several discussions arising from a variety of conceptual understandings [54]. Although the sample size posed some limitations, the richness in the participants’ descriptions was a strength, thus contributing to enhancing the information power [55]. Another limitation may be related to the researchers not being able to conduct member checks to improve the credibility of the data. For practical reasons, it was impossible to gather the same sample of students to validate their statements. However, during the focus group interviews, the participants were asked to provide detailed answers and were given the necessary time to reflect and express their experiences, thus confirming and or disagreeing with each other’s perceptions. Furthermore, potential research biases should be acknowledged given that the data collection and analysis were conducted by all researchers who were nurse educators employed at the same university as the students, hence entailing a prior understanding of the research context. However, the researchers were not involved in the students’ inspiration practice period, which may have limited the research bias regarding data collection. Another limitation may be its specific theoretical framework [37]. We are aware that other researchers, by using another theoretical framework, would probably discuss the findings accordingly and, hence, interpret the findings differently.

## Conclusion

To the best of the researchers’ knowledge, this is the first study exploring first-year nursing students’ experiences with one week of inspiration practice at a nursing home by employing peer mentoring as a teaching and learning method. The findings revealed that first-year students were inspired by their senior peers to keep learning and moving forward. By being close to their mentors and having confidence in their professional knowledge and teaching and supervision methods’, learning was easily transferred from the third-year students to first-year students. Moreover, person-related, teaching-related and situation-related factors were perceived as drivers that positively influenced students’ learning in nursing homes.

The findings have indicated that first-year students had both positive and less positive experiences with attending a one-week inspiration practice at nursing homes. The challenges with inspiration practice were related to situation-related learning transfer factors, such as clinical field not providing enough placements; therefore, the third-year students had to improvise and be creative. However, despite some challenges, mentorship during the one-week inspiration practice offered significant advantages to both mentors and mentees. To fully harness these advantages, we recommend that first-year educational programmes implement person-centred care for older people into the educational curriculum. This should include a one-week compulsory inspiration practice placement in settings exclusive to older people, such as nursing homes. Moreover, peer mentoring as a teaching and learning method, with themes especially designed to focus on nursing and caring for and with older people, offers first-year students insights into nurses’ roles and responsibilities at nursing homes. We believe that such a programme can prevent ‘reality shock’, reduce dropout rates, enhance academic achievements and cultivate personal and professional qualities in students at all levels of their education programmes. More research is needed to explore how peer mentoring is experienced by students enrolled at different levels of Bachelor of Nursing Education and may contribute to their preparation to care for older people in nursing homes.

## Data Availability

The datasets used and/or analysed during the current study are available from the corresponding author upon reasonable request.

## Abbreviations

P: Participant (followed by a number indicating the number of participants in the focus group)
FG: Focus Group (followed by a number indicating the number of the focus group)
